# Effects of Gabapentin on the Treatment of Behavioral Disorders in Dogs: A Retrospective Evaluation

**DOI:** 10.3390/ani14101462

**Published:** 2024-05-14

**Authors:** Taylor Kirby-Madden, Caitlin T. Waring, Meghan Herron

**Affiliations:** 1 Massachusetts Veterinary Behavior, Dennis Port, MA 02639, USA; 2 Gigi’s, Columbus, OH 43110, USA; mherron@gigis.org

**Keywords:** gabapentin, effectiveness side effects, veterinary, anxiety, phobia, arousal, global fear, aggression, survey, psychopharmacology

## Abstract

**Simple Summary:**

Gabapentin is a medication often prescribed to dogs for some types of pain and/or behavioral disorders. This study surveyed dog owners regarding the effectiveness and side effects of gabapentin when it was prescribed for their dogs with problem behaviors. According to the owners in this study, gabapentin was well-tolerated with minimal side effects in most dogs. Few reported side effects were considered bothersome, and none were a cause for discontinuation of the medication. The most common reason owners gave for discontinuation of gabapentin was perceived lack of effectiveness. Sedation was the most commonly reported side effect, mostly when given at high doses. Close to one-third of owners reported that their dogs displayed no unwanted side effects, even at the highest doses. Overall, gabapentin appears to be a safe and well-tolerated medication choice for dogs with behavior disorders. Specific dose ranges (milligram per kilogram of body weight) did not correlate with reports of side effects nor effectiveness, suggesting that some dogs may be more sensitive or resistant to adverse and/or therapeutic effects than others and multiple dosage trials may be needed before finding the best fit.

**Abstract:**

The use of gabapentin in treating dogs with behavioral disorders is not well described. To characterize behavioral effects of gabapentin, this study surveyed 50 owners whose dogs were prescribed gabapentin at a veterinary behavior-focused practice over a five-year period. Most owners (72%) reported that gabapentin was moderately or very effective at improving their dog’s behavior. The majority of owners reported at least one side effect (70%), with sedation being the most common. Sedation was more likely to be seen at doses higher than 30 mg/kg. Specific dose ranges (mg/kg) did not correlate with any other reports of side effects nor effectiveness. Dogs with a diagnosis of conflict-related aggression were more likely to have owners report that gabapentin was effective at improving behavior compared to dogs with other behavioral diagnoses (*p* = 0.04), while dogs diagnosed with aggression secondary to high arousal were less likely to have owners report that gabapentin was effective (*p* = 0.01). Overall, reports of effect varied widely and, with the exception of sedation, did not correlate with specific mg/kg dose ranges. Results suggest that some dogs may be more sensitive or resistant to adverse and/or therapeutic effects than others and multiple dosage trials may be needed before finding the best fit.

## 1. Introduction

Behavioral disorders in dogs can pose both safety and welfare concerns for dogs and the humans that care for them. Owners managing their dogs’ behavioral disorders report that these behaviors diminished the well-being for both themselves and their pets [1,2], putting these dogs at risk of relinquishment or euthanasia [3,4]. Veterinarians should, therefore, reach for interventions that provide swift relief of symptoms and management plans that take both the pet and human well-being into consideration.

Gabapentin is a fast-acting medication commonly used to treat behavioral disorders in dogs, although efficacy and potential for side effects are not well described in the literature [5]. Gabapentin’s behavioral effects are likely due to its binding to subunits alpha-2-delta of calcium channels, thereby inhibiting calcium influx into neuronal cells and reducing the release of various monoamine neurotransmitters like noradrenaline and glutamate [6,7]. Onset of effect is approximately 30 to 90 min and duration of effect is 7–8 h on average [8,9]. Gabapentin can increase GABA synthesis and, in humans, increases serum serotonin levels in whole blood [10]. While gabapentin initially gained FDA approval to treat neuropathic pain, spasticity, and seizures in humans, it is now also used as an adjunct therapy to treat a variety of psychiatric disorders, including bipolar disorder, anxiety disorders, and insomnia [11]. In veterinary medicine, gabapentin is used in dogs to treat chronic and post-operative pain [12,13], seizures [14], and behavioral disorders including fear associated with veterinary clinic visits [15], storm phobia [16], and canine compulsive disorder [17].

From a psychopharmacology standpoint, gabapentin offers versatility in its dosing. Gabapentin can be prescribed as an as-needed, immediate, and short-acting medication given 90 min prior to anticipated stressful events and repeated once or twice per day, depending on the frequency of the stressor (event use), and/or as an everyday medication given every eight to twelve hours (daily use) [18]. Additionally, gabapentin can also be used as part of a daily or as-needed poly-psychopharmacologic therapy plan in combination with other psychoactive medications, such as longer-acting selective serotonin reuptake inhibitors (SSRIs), tricyclic antidepressants (TCAs), or serotonin and noradrenaline reuptake inhibitors (SNRIs) [19]. Starting doses vary based on severity of clinical signs, urgency for relief of signs, and extent to which sedation is desired or unwanted in each circumstance. Clinicians typically prescribe an initial trial dose on the mid-to-lower end of the published dose range (10–15 mg/kg) for behavioral use. If the owner notes no relief of symptoms after the initial trial dose(s), they are instructed to increase the dose in gradual increments, titrating up until they see the desired therapeutic effect in their pet without excess sedation. When using gabapentin as part of a poly-psychopharmacologic therapy, most clinicians recommend starting the dosing trial either before the start of additional medications or only once other psychoactive medications are established and stable in the pet’s system [20].

Although the recommended standard dosage range of gabapentin is 10–30 mg/kg [18], veterinary psychopharmacology formularies and published case reports describe its use at higher ranges [15]. Stollar et al. found that a 50 mg/kg oral dose of gabapentin prescribed for event use prior to veterinary visits was well-tolerated in dogs without major adverse effects. Gabapentin prescribed for dogs for daily use to treat chronic pain has been utilized at doses up to 500 mg/kg/day with only 10% of patients experiencing adverse side effects, sedation being the most commonly reported [12]. The use of gabapentin as a regular, daily mono- or poly-psychopharmacologic medication has not been well described in the veterinary literature and little information exists regarding the potential for both beneficial and/or adverse behavioral effects at doses above 30 mg/kg. This study attempts to characterize both the desired and unwanted owner-reported effects of gabapentin, including doses above 30 mg/kg in dogs presenting to a behavior practice, whether prescribed for event use, daily use, or both. Given the findings of Platt et al., Stollar et al. and Davis et al. [12,14,15], we hypothesized that side effects, particularly sedation, may be seen at the higher doses but that this effect would not bother most owners. Based on anecdotal reports, we also hypothesized that older dogs would be more likely to have sedation and ataxia reported than younger dogs.

## 2. Materials and Methods

### 2.1. Animals

A review of patient files for dogs seen at a veterinary behavior-exclusive private practice on Cape Cod, MA, USA (Massachusetts Veterinary Behavior), from 1 January 2018, to 1 June 2023, was conducted. Electronic medical records (Vetter software, version 1) for dogs prescribed gabapentin to treat a behavioral disorder were reviewed to collect pertinent owner (name and email contact) and dog (date seen, age, breed, sex, weight, behavioral diagnoses) information. Diagnoses that warranted inclusion in this study included fear-related aggression (aggression triggered by fear of an unfamiliar person or dog), conflict-related aggression (aggression towards an owner that is triggered by a perceived threat to personal space or loss of a resource), aggression secondary to high arousal (aggression where the target of the aggression is not the primary trigger for it, but rather another event or arousing/frightening/threatening stimuli in the environment is), global fear (fear in response to a wide range of environmental, particularly novel, stimuli), generalized anxiety (persistent anxiety, vigilance, and/or reactivity that is not context-specific), separation anxiety, noise phobia, and other specific phobias (e.g., cars) [21]. Records were closely reviewed for information regarding gabapentin dose and instructions, as well as other psychotropic medication(s) prescribed at the time of the behavior-focused visit and/or previously prescribed by the referring veterinarian and still being administered at the time gabapentin was prescribed. Files for dogs whose owners had elected euthanasia or rehoming for their dog were excluded from analysis, as were files for dogs whose owners never administered gabapentin, and dogs who were prescribed gabapentin for non-behavior related disorders (pain, neuropathy), even if there was a comorbid behavioral indication for gabapentin. Selection criteria did not exclude dogs who were on other psychoactive medication(s). It was standard for the prescribing clinician in this practice that when gabapentin was prescribed as part of a poly-psychopharmacologic therapy, owners were instructed to start only one medication at a time so that its effects (both desired and unwanted) were not conflated with other medications. When available, data were verified with the medical records on file by members of the study team who were blinded to the owner survey responses.

Owners of dogs who met the inclusion criteria were sent an introductory email offering the opportunity to participate in a voluntary survey about their dog (See Supplementary Material). This email included a link to the gabapentin survey which took an estimated 10–15 min to complete. An electronic informed consent release detailing the purpose of the study and that their participation was voluntary and confidential was obtained for each participant prior to completing the survey. Clients who did not respond within two weeks were then texted an invitation to participate; no further follow-up was attempted.

### 2.2. Survey

The survey included a combination of free text and closed-ended multiple choice and Likert-type scale questions. Since gabapentin is most commonly prescribed as an initial trial dose that is then titrated up to the desired therapeutic effect, the survey aimed to capture the full range for each individual dog. Owners were asked questions that confirmed the lowest and highest doses of gabapentin given to their dogs (referred to for analysis later as the qualitative “lowest dose” and “highest dose”, or just as the qualitative “lowest dose” when only one dosage was given). This allowed for each dog to have a reported “lowest dose” and “highest dose” in relation to themselves. The “lowest” and “highest” doses were also later calculated into quantitative mg/kg categories for statistical analysis and inter-dog comparisons.

Participants were then asked to characterize how they administered gabapentin. For example, did they give it to their dog only as needed just prior to a stressful event (event use), or did they give it every day (daily use)? In cases when owners gave gabapentin daily, but also gave an additional or higher mg dose as needed for events, they could select that they utilized the medication for their dog in both manners. A series of questions regarding the reported “lowest” and “highest” doses given followed, including whether or not the owner continued the use of gabapentin after the first time trialing the medication, owner’s perception of how effective the medication was at improving their pet’s behavior, noted side effects (and if associated with lowest dose, highest dose, or both), whether side effects were considered bothersome and, if discontinued, reasons for doing so. Owners who reported discontinuing gabapentin were prompted to choose from the following reasons for doing so: “lack of efficacy”, “no longer needed”, and “undesirable effects”; owners were able to choose more than one reason for discontinuation as well as to provide additional responses in the free text area.

For owner perception of efficacy, owners were asked to rate how effective gabapentin was at improving their dog’s behavioral problems on the following Likert-type scale: “very effective”, “moderately effective”, “slightly effective”, or “not effective”. These categories were collapsed for statistical analysis as “effective” (“very effective”, “moderately effective”) or “minimally/not effective” (“slightly effective”, “not effective”).

Potential side effects listed in the survey were “sedation” (defined in the survey as “your dog was tired/sleepy from this medication”), “increased appetite”, “unsteady or drunk” (defined as “falling, stumbling, bumping into walls, etc.”; henceforth referred to as “ataxia” for analysis), “increased activity” (defined as “running or jumping around, as though your dog just had a very strong cup of coffee”), “agitation” (defined as “seemed irritable, restless, had difficulty relaxing, vocalized”), “vomiting”, “diarrhea”, “constipation”, “urinary dribbling” (henceforth referred to as “urinary incontinence” for analysis), “new aggression” (defined as “your dog snapped, growled, lunged, or bit people or other animals while under the influence of this drug when your dog had never previously shown aggression in any context”), and “increased aggression” (defined as “your dog snapped, growled, or bit people or other animals in a manner that is very uncharacteristic for this dog when not under the influence of this medication”). For reported side effects, owners were prompted to then report how much that side effect bothered them as “very much”, “somewhat”, “not at all”, or “not applicable”.

### 2.3. Statistical Analysis

Data were entered into an Excel database (Microsoft Corp., Redmond, WA, USA) and exported and analyzed using Intercooled Stata version 17.0 (Stata Corp., College Station, TX, USA). Descriptive statistics were computed for all variables. Blank responses were excluded from analyses.

Using data distribution parameters (e.g., tertiles), the following continuous variables were categorized for statistical purposes: dog age (<1.7 y, 1.7–4.0 y, >4.0 y) and “lowest” and “highest” gabapentin doses for each dog (<15.0 mg/kg, 15.0–30.0 mg/kg, >30.0 mg/kg). The “highest dose” group was further categorized to investigate higher dose ranges (0–15 mg/kg, 15–30 mg/kg, 30–49 mg/kg, and >50 mg/kg). Dog weight was categorized as <10 kg, 10–25 kg, >25 kg to best investigate data distribution and interest in low weights. Binomial exact 95% confidence intervals were calculated for the proportion of dogs with owner-reported side effects at their “lowest” and/or “highest” doses of gabapentin. Associations between categorical variables were assessed using the Pearson chi-square test or, if any expected cell value was ≤1 or 20% or more expected cell values were ≤5, Fisher’s exact test. The exact McNemar chi-square test was used for comparing paired observations (at the dog/owner level) between categorical variables (i.e., associations between reported adverse effects for lowest dose as compared to highest dose gabapentin). Statistical significance was based on a *p*-value ≤ 0.05.

## 3. Results

The initial file review identified 253 owners whose dogs met the inclusion criteria. Email invitations for the survey were sent to all 253 people with follow-up text message reminders sent five days later. A total of 84 owners submitted surveys (response rate of 33%). Thirty-four surveys were rejected due to incomplete responses, leaving a total of 50 completed surveys for analysis.

### 3.1. Dog Demographics

Of the dogs in the study, thirty-one were castrated males (62%), twelve were ovariohysterectomized females (24%), four were intact males (8%), and three were intact females (6%). Twenty-eight were mixed breed dogs (56%) and twenty-two were purebred/purebred hybrid dogs (44%, see Appendix A). The mean age at the time that gabapentin was first prescribed was 3.4 years (SD = 2.8; range five months to 12.7 years). Mean body weight of the dogs as recorded in the medical records was 23.9 kg (SD = 13.2; range 6.8–84.0 kg).

### 3.2. Behavioral Diagnoses and Concurrent Psychopharmacological Therapy

Behavioral disorders for which gabapentin was prescribed included fear-related aggression (*n* = 37), generalized anxiety disorder (*n* = 34), specific phobia other than noise (*n* = 22), conflict-related aggression (*n* = 15), noise phobia (*n* = 9), separation anxiety (*n* = 8), aggression secondary to high arousal (*n* = 7), and global fear (*n* = 6). Comorbidities were common and all dogs had more than one disorder diagnosed. All behavior diagnoses were considered during statistical analysis.

Forty-eight dogs were prescribed gabapentin as part of a poly-psychopharmacologic therapy. Seventeen dogs were also prescribed, already receiving, and/or weaning off one or more daily, long-acting medication at the start of or during treatment with gabapentin (fluoxetine, *n* = 22, sertraline, *n* = 12, venlafaxine, *n* = 6, paroxetine, *n* = 5, clomipramine, *n* = 4). Thirty-six dogs were prescribed, already receiving, and/or weaning off at least one or more additional, immediate, short-acting, event medication at the start of or during treatment with gabapentin (trazodone, *n* = 19, clonidine, *n* = 15, acepromazine, *n* = 2, alprazolam, *n* = 2, lorazepam, *n* = 2, dexmedetomidine gel, *n* = 1).

### 3.3. Dosing and Administration Reports

Most owners (*n* = 43, 86%) confirmed the administration of at least two dosages, as qualitatively described on the survey as their dog’s “lowest” and “highest” doses of gabapentin. Some owners (*n* = 7) only gave gabapentin at one “lowest” dose and never trialed the medication at a higher dose. The mean “lowest” dose reported for all dogs in the study was 11.7 mg/kg (SD–5.5; range 3.0–25.4 mg/kg). The mean “highest” dose administered was 37.8 mg/kg (SD = 13.4; range 13.0–64.9 mg/kg). Eight owners (18%) reported that their “highest” administered dose of gabapentin was ≥50 mg/kg. Several dogs had qualitative “lowest” dosages that were quantitatively (based on mg/kg) higher than other dog’s qualitatively report “highest” doses and vice versa.

Most owners reported that they gave their dogs gabapentin in a daily use manner (*n* = 34, 68%), whereas some used this medication only for anticipated stressful event use (*n* = 9, 18%). A smaller number (*n* = 3, 6%) of owners reported daily use at a lower dose and event use at a higher dose for their dogs. A few respondents (*n* = 4, 8%) reported that they only gave gabapentin 1–3 times. Most owners were still administering it to their dog at the time of the survey (*n* = 36, 72%), while fewer were no longer administering it (*n* = 14, 28%). Of the fourteen owners who stopped giving the medication, the reasons for doing so were “lack of efficacy” (*n* = 11), “no longer needed” (*n* = 2), and “undesirable effects” (*n* = 2). For dogs whose owners discontinued gabapentin due to “lack of efficacy” (*n* = 11), their mean “highest” dose was 34 mg/kg (SD 17.1 mg/kg), with a median 35.3 mg/kg (range 9.4–58.4 mg/kg).

Descriptions regarding therapeutic effects were reported by owners as “very effective” (40%, *n* = 20), “moderately effective” (32%, *n* = 16), “slightly effective” (8%, *n* = 4), or “not at all effective” (20%, *n* = 10). There was no association between owner-reported effectiveness (effective vs. minimally/not effective) and dosage for either the “lowest” or “highest” dose categories (*p* = 1.0 and *p* = 0.5, respectively).

### 3.4. Side Effects

Thirty percent of owners reported that their dog showed no side effects as a result of gabapentin administration at any dose. Most owners did report at least one side effect at either or both of their dog’s “lowest” or “highest” doses, with sedation being the most commonly reported (*n* = 23, 46.0%), followed by agitation (*n* = 12, 24%) (Table 1). Age (categorized) and body weight (categorized) were not associated with a greater likelihood of side effects (all *p* > 0.2). All owners who reported increased activity as a side effect (*n* = 7) also reported agitation as a side effect, but not all owners who reported agitation (*n* = 12) reported increased activity.

When comparing side effects noted after giving the qualitatively reported “lowest” dose in relation to the qualitatively reported “highest” dose, more owners reported side effects after administering their dog’s “highest” dose (*p* < 0.0001). Of the listed side effects, sedation (*p* < 0.0001) and ataxia (*p* = 0.004) were more likely to be associated with qualitatively reported “highest” doses than they were with reported “lowest” doses.

Of the owners reporting sedation (*n* = 23), most reported that seeing this side effect in their dog did not bother them at all [*n* = 15 (65.2%)] or only somewhat bothered them [*n* = 7 (30.4%)]. Only one of these owners (4.3%) reported being very bothered by sedation as a side effect. Seven out of the nine (77.8%) owners who reported ataxia were at least somewhat bothered by this side effect, with three of them (33.3%) being “very bothered”. All of the owners reporting agitation (*n* = 12) were bothered by it with seven owners (58.3%) being “somewhat” bothered and five owners (41.7%) being “very bothered”. Of the seven owners reporting increased activity, four (57.1%) were “very bothered” by it and one (14.3%) was “somewhat bothered”. The three owners who reported new aggression reported that it bothered them “very much” and none of these owners reported giving a higher dose. The single owner reporting increased aggression was only “somewhat bothered” by this side effect and did not report any new aggression. All owners reporting new aggression and all owners reporting increased activity also reported agitation as a side effect. Of the three owners reporting an increased appetite, only one owner was “very bothered” by it and the other two were not bothered at all. Although only two owners reported diarrhea as a side effect, both were “somewhat bothered” by it. In contrast both owners reporting urinary incontinence were “very bothered” by it.

When evaluating the data based on a quantitative, calculated mg/kg dose at both the reported “lowest” dose and “highest” dose, reported side effects were quite varied. Table 2 shows side effects at each quantitative mg/kg dose category according to the dogs qualitatively reported “lowest” administered dose. No “lowest” dose mg/kg dosing category was more likely than another to be associated with owner reported side effects. Similarly, no “highest” dose mg/kg dosing category was more likely than another (<15 mg/kg, 15–30, 30.1–49.9, ≥50 mg/kg) to be associated with owner-reported side effects for dogs (Table 3). However, when comparing rates of reported side effects above or below 30 mg/kg, sedation was significantly (*p* = 0.05) more likely to be reported as a side effect in dogs given doses >30 mg/kg [*n* = 18 of 31 (58%)] than dogs given doses ≤30 mg/kg [(*n* = 3 of 12 (25%))].

Analysis of behavioral diagnosis/es and owner-reported effectiveness of gabapentin revealed two significant associations (Table 4). First, dogs with a diagnosis of “conflict-related aggression” were more likely to have reports from their owner that gabapentin was “effective” at improving their dog’s behavior compared to owners of dogs diagnosed with other conditions (93% vs. 63%, respectively; *p* = 0.04). Conversely, dogs with a diagnosis of “aggression secondary to high arousal” were less likely to have owners report that gabapentin was “effective” at improving their dog’s behavior compared to owners of dogs diagnosed with other conditions (29% vs. 79%, respectively; *p* = 0.01). No other diagnoses had significant associations with owner reports of effectiveness.

## 4. Discussion

Overall, gabapentin seemed to be well-tolerated by most dogs with close to one-third of owners reporting zero side effects. Not surprisingly, sedation was the most commonly reported side effect, particularly at the qualitatively reported “highest” doses (mean 37.8 mg/kg). Contrary to one of our initial hypotheses, an age effect was not appreciated. The frequency of sedation as a reported side effect matches previous reports in both dogs and cats, demonstrating common sedative effects of this drug [14,15,22]. The mean of the qualitatively reported “lowest” doses (11.7 mg/kg) administered in this study population is similar to the mean dose of 10 mg/kg reported by Platt et al. [14]. However, Platt et al. reported a sedation rate of 45% for these dogs which is substantially greater than the reported sedation rate of 4% for dogs in the current study’s “lowest” reported dose. Reported sedation rates in the Platt et al. study (46%) more closely match the reported sedation rate of 41.6% for dogs in the current study’s qualitatively reported “highest” dose (mean 34 mg/kg). It is worth noting that Platt et al. reported on the treatment of dogs with refractory idiopathic epilepsy, all of whom were on other anticonvulsants that may have contributed to sedative effects. That said, nearly all dogs in the current study were on concurrent psychoactive medications with the potential to increase sedation. One explanation for the vast difference in reports of sedation at lower mg/kg dosages between these two studies is that dogs with behavioral disorders may be more likely than dogs with epilepsy to override sedative effects of medications due to chronic or repeated sympathetic nervous system and Hypothalamic-pituitary-adrenal (HPA) axis activation associated with anxiety and fear-based disorders [23,24,25,26].

While sedation is commonly reported at doses above 30 mg/kg, both in the current study and others, it was seldom reported to be bothersome, and sedation was not listed as a reason for discontinuing gabapentin by any owners. Tolerance of sedation may be due to it being a desired behavioral effect in many dogs in this study population. Anxiety and fear-related behavior disorders often lead to frenetic, at times hyperkinetic, behavior displays, including pacing, panting, jumping, scanning of the environment and attempts to flee or escape, as a means of coping with a present or anticipated threat, whether real or perceived [27,28]. A sedative effect would likely lead to a reduction in these clinical signs, thereby fundamentally recategorizing sedation as a desirable effect rather than an adverse effect in these patients. We suggest that for at least some patients with hyperexcitability or excessive activity that the “sedative” effects may in fact allow the dog to more closely approximate normal canine activity levels.

Agitation was the second most commonly reported side effect and was noted in all dose ranges, including those lower than 15 mg/kg. Interestingly, all of the owners reporting increased activity also reported agitation. These findings suggest that increased activity is likely a manifestation of agitation and the two side effects clearly have some overlap. Stollar et al. describe a rate of 5% for “increased activity” as a side effect of gabapentin when given as a pre-veterinary appointment, but other studies do not report on agitation specifically [15]. Reports of agitation and increased activity across multiple dosing ranges may be a result of paradoxical hyperexcitability—an adverse reaction to a medication that involves an increase in motor activity, excitability, and potential towards irritability. Paradoxical drug reactions are uncommon events associated with most psychoactive medications in humans and dogs [29,30]. Herron et al. described agitation and increased activity as side effects in dogs across all dose ranges of diazepam, including several at the lowest end of the dose range [31]. While agitation is not a common side effect of most psychoactive medications, the potential exists, and gabapentin is no exception. Given that all owners reporting agitation were bothered by it, regardless of dose ranges, adjustment in dose may not be helpful for these patients and a medication change is likely warranted.

While ataxia was noted as a side effect by some owners at their dog’s qualitatively reported “highest” dose, it was not a commonly reported side effect overall. These findings are similar to previous studies’ findings that describe the use of gabapentin to treat behavior disorders in dogs. Stollar et al. reported only 9% and Bleur-Elsner reported 16.7% of dogs showing ataxia as a result of gabapentin treatment, including dogs receiving doses above 50 mg/kg [15,16]. Case reports in the human psychiatric literature have suggested that some individuals may possess a high cerebellar affinity for gabapentin binding that can increase ataxia at “initial” dose ranges [32]. The subsequent result of muscle relaxation and/or incoordination, especially when profound, is a dog who wobbles when ambulating, sometimes falling or collapsing. Veterinarians prescribing gabapentin, or any other medication with ataxic effects, should caution owners to block access to stairs or elevated furniture until the effects in that individual patient can be ascertained regarding fall risks.

Few other side effects were reported, which is similar to previous studies [15,33]. While increased aggression and/or new reports of aggression were uncommon, such side effects were problematic for all owners reporting them. Perhaps this study population selected for dogs with a greater propensity for aggressive behavior, considering most of them presented with an existing aggression issue. The patients that did not present with aggression had an underlying fear or anxiety-related disorder severe enough to warrant referral to a veterinary behavior practice. Dogs with profound anxiety may be prompted to display aggression in response to triggers at a lower intensity of provocation than dogs without anxiety [21], regardless of gabapentin use. It is also possible, at least for the dogs in this study, that the aggression was related to agitation, as all dogs reported to have new aggression were also reported to have agitation as a side effect. While our results cannot speak to any causation, dogs who are agitated could be more sensitive to triggers, just as a dog with anxiety, as described above. It is also worth noting that we did not ask owners how many times a specific dose was trialed. It is possible that owners may have misattributed aggression and agitation as an effect of gabapentin rather than being triggered by an environmental stressor; having owners trial a particular dose multiple times would have made this clearer but would have also posed a significant safety and welfare concern if the new aggression was truly a gabapentin-induced effect. Idiosyncratic reports of increased aggression and/or disinhibition of aggression exist with many psychoactive medications [31,34,35], and gabapentin could simply be among them. Clinicians, therefore, should be mindful of any potential increase in aggression risk when prescribing psychoactive medications.

Reports of sedation and ataxia correlated with qualitatively reported “highest” administered doses of gabapentin, compared to the qualitatively reported “lowest” doses. Lack of sedation and ataxia in the “lowest” dose may be why most owners were willing to trial a higher dose at subsequent dosing trials. That said, with the exception of ataxia not being reported at doses below 15 mg/kg, reports of sedation and ataxia spanned all other mg/kg dose ranges.

Owner perceptions of effectiveness were reported similarly for both the qualitatively reported “lowest” and “highest” doses. This may be due to the substantial overlap between “lowest” and “highest” dose ranges on a mg/kg basis. For example, what was reported to be the “highest” dose for one dog was in several instances quantitatively lower than another dog’s “lowest” dose and vice versa. That said, when looking at the data in a quantitative mg/kg basis, no dose range category was more likely to be correlated with owner reports of effectiveness, either. These results may be due to the fact that effectiveness is a subjective term and open to owner interpretation. All owners were aware they were giving gabapentin, and this may have influenced their perception of its effectiveness. In other words, the “placebo effect” may have influenced owner perceptions and without a control group true efficacy cannot be garnered from the data presented here. Furthermore, the survey did not specify the effectiveness of improving a particular behavior or problem. Dogs with multiple comorbidities may have improved in some aspects of their behavior but not in others, leading some owners to respond differently. A placebo-controlled study limited to specific behavioral diagnoses would describe the efficacy of gabapentin to a clearer extent. That said, placebo-controlled studies in a population of aggressive dogs would face several ethical barriers, including safety and welfare concerns for both the patient and the humans caring for them.

Another potential reason this study had difficulty correlating effectiveness, as well as most side effects, with specific mg/kg dose range categories is that gabapentin’s clinical effect has substantial individual variability, regardless of dose. Response to gabapentin may be mediated by genetic heterogeneity within populations; rat and human studies have found varying efficacy based on polymorphisms [36,37]. Pharmacogenomics, or the study of individual human and/or ethnic variation on gene expression involved in drug transporters, metabolism, and receptors, is a growing area of study within the field of pharmacokinetics; it would be reasonable to speculate that our genetically distinct breeds and types of dogs may experience similar heterogeneity [38]. The variability of both effectiveness and side effect frequency in dogs as a result of gabapentin administration may be modulated by basic genetic differences. Effect may also be altered by renal health, as a decrease in function has been correlated with a higher concentration of gabapentin in other mammals [39,40]. Renal function data was not collected in this population, although all owners were instructed to obtain yearly clinical chemistry analysis from their primary care veterinarian. Gastrointestinal disease may also affect absorption and, therefore, propensity for both therapeutic and side effects in dogs. Gabapentin is water-soluble and GI tract absorption occurs via the l-amino acid transport system in the proximal small intestines [41]. Early bioavailability studies suggest that absorption may be capacity-limited, preventing some patients from responding to higher doses [42]. One could also infer that upper GI disease and concurrent medications could affect absorption and subsequent bioavailability. Future studies may investigate the relationship between renal and GI health and reported effects of gabapentin—both from a therapeutic and adverse effect standpoint.

Most owners report that gabapentin was effective at improving their dog’s behavior. Of particular interest was that owners of dogs with a diagnosis of conflict-related aggression (CRA) were more likely to rate gabapentin as being effective than were owners of dogs with other diagnoses. CRA is characterized as an affective aggression directed towards familiar household humans when a dog perceives they are a threat to their personal safety and resources. This type of aggression often presents as body-handling sensitivity, as well as an intolerance of being approached when resting and in possession of a valued item. A potential increase in physical comfort as a result of gabapentin’s effects on neuropathic pain [43] may have led to a greater tolerance of body handling and the approach of family members when the dog was in a somewhat vulnerable, resting state. Inversely, owners of dogs with a diagnosis of aggression secondary to high arousal (ASHA) were more likely to rate gabapentin as being ineffective at improving their dog’s behavior, compared to owners of dogs with other diagnoses. ASHA is precipitated by sudden or unexpected external sources (loud noises, social stimulation) that cause high emotional intensity and a rapid transition into aggression directed towards targets in the proximal environment who, were it not for the sudden stressor, might not otherwise be triggers [21]. Different types of aggression appear to be mediated and/or activated by different subcortical brain regions [44]. It may be that gabapentin’s pharmacological effects do not adequately prevent sharp spikes in the sympathetic nervous system arousal driving ASHA.

Almost all dogs described in this study were currently taking, being weaned from, or prescribed at least one additional psychoactive medication at the time gabapentin was administered. While an ideal study would measure the effects of gabapentin as a standalone therapy, such use is uncommon in a behavior-focused practice. At the referral level, practices limited to behavior see cases with greater severity and urgency than one might see in a general practice setting [45]. Often safety and welfare concerns warrant immediate and multi-modal therapies, making polypharmacy an essential part of the treatment plan. As the goal of the study was to capture and characterize the effects of gabapentin in this specific population of dogs, it seems reasonable to include and accept the results as such. Concurrent psychoactive treatment was not further analyzed for likelihood of side effects or effectiveness as the number of dogs receiving each concurrent medication was small and variable. Future studies may investigate the efficacy of gabapentin given with specific psychoactive medications.

There were several limitations to our study, the most apparent being sample size. Given the incidence of comorbidities and wide dose range utilized, a larger sample size might better elucidate what if any factors might correlate with a greater therapeutic effect or potential for side effects. The study population described here, while smaller than ideal, was still adequately representative of dogs who visit a veterinary behavior-focused practice. Males outnumbered females 2:1, which matches previously reported data for dogs presenting to veterinary behavior practices [46,47,48]. Most dogs were under 4 years of age at the time of presentation to a veterinary behavior-focused practice. This is consistent with Anderson et al. and Bamberger et al. [49,50], and correlates with published data showing behavior problems being the top reason for euthanasia of dogs 3 years of age and younger [27,50,51]. Dogs included in this study had multiple behavioral comorbidities, with fear-related aggression and generalized anxiety being the most common. The high number of dogs with fear-related aggression may correlate to the general caseload of dogs presenting to a veterinary behaviorist as it has historically been the top presenting complaint for this specialty [49]. As fear-related aggression cases are often referred, rather than treated by general practitioners, a survey of the use of gabapentin in a general practice population might yield different results.

To our knowledge, this is the first paper describing the side effects and effectiveness of gabapentin as a daily adjunct and/or an event medication for behavioral disorders in dogs [52]. Other published gabapentin papers describe its use for specific, temporal events but not as a daily medication for long-term treatment of behavioral disorders, and they do not characterize side effects or owner perceptions of those effects. Gabapentin pain studies are more common than behavior studies, but, with the exception of Davis et al. [12], the gabapentin dosing for pain is lower (10–20 mg/kg) than dosing typically used for behavioral disorders [43,53,54]. The Davis et al. study on gabapentin prescribed for dogs with chronic pain published data on dosing of up to 500 mg/kg/day (250 mg/kg BID), yet the authors reported only 10% of patients experienced sedation. This may be due to the Davis et al. study being a medical records review rather than an owner survey. Sedation may, in fact, have been reported more frequently were owners specifically surveyed about it. Many owners may have either not found it to be bothersome and/or not considered it worthy of reporting to a medical professional without prompting. Still, Davis et al.’s dose range for gabapentin is far greater than what is commonly used in specialty and/or general practice and should perhaps prompt practitioners to consider higher doses for patients not experiencing adverse effects when greater therapeutic effect is needed.

## 5. Conclusions

The results of this study suggest that gabapentin may be a beneficial treatment for dogs with behavioral disorders, particularly those with aggression towards familiar humans in the household. Sedation appears to be a common side effect at doses higher than 30 mg/kg, yet it has not been shown to be problematic nor a reason to discontinue the medication. Other side effects, such as ataxia and agitation, are less common and do not seem to be dose-related. Owners find gabapentin to be effective at improving their dogs’ problem behaviors at both high and low dosages. The vast dose range for both therapeutic and unwanted effects suggests that clinicians may need to guide their clients through multiple dose trials before finding a dose that works best for their pet. Individual patient responses must be carefully assessed and monitored for the best clinical results. Clinicians strive to provide therapies that are effective at improving canine behavioral disorders without bothersome side effects, and this study suggests gabapentin may be a good choice for many dogs.

## Figures and Tables

**Table 1:** Frequency of side effects based on reported doses of gabapentin administered to 50 dogs with behavioral disorders. Note these categories of dosing are qualitative, based on how owners responded when asked about their dog’s side effects after receiving the “lowest” and “highest” doses they administered and are not based on a mg/kg calculation.

Side Effect	No. Dogs at “Lowest” Dose Alone (*n* = 50) ^1^	No. Dogs at “Highest” Dose Alone (*n* = 43) ^2^	No. Dogs at Both Doses (*n* = 43) ^2^	Total No. Dogs (*n* = 50); 95% CI	*p*-Value ^3^
Sedation	2 (4.0%)	18 (41.9%)	3 (7.0%)	23 (46.0%; 31.8–60.7%)	<0.0001
Ataxia	0	9 (20.9%)		9 (18.0%; 8.6–31.4%)	0.004
Increased activity	3 (6.0%)	3 (7.0%)	1 (2.3%)	7 (14.0%; 5.8–26.7%)	1.0
Agitation	4 (8.0%)	6 (14.0%)	2 (4.7%)	12 (24.0%; 13.1–38.2%)	0.1
Increased appetite	0	2 (4.7%)	1 (2.3%)	3 (6.0%; 1.3–16.5%)	0.5
Vomiting	0	0	0	0	NP
Diarrhea	0	2 (4.7%)	0	2 (4.0; 0.5–13.7%)	0.5
Constipation	0	1 (2.3%)	0	1 (2.0%; 0.05–10.6%)	1.0
Urinary incontinence	0	1 (2.3%)	1 (2.3%)	2 (4.0%; 0.5–13.7%)	1.0
New aggression	3 (6.0%)	0	0	3 (6.0%; 1.3–16.5%)	0.5
Increased aggression	0	1 (2.3%)	0	1 (2.0%; 0.05–10.6%)	1.0
One or more of the above	6 (12.0%)	22 (51.2%)	7 (16.3%)	35 (70.0%; 55.4–82.1%)	<0.0001

^1^ Some of the dogs exhibiting side effects at only their “lowest” dose only received a “lowest” dose (i.e., no “highest” dose provided to the dog): sedation (*n* = 2), increased activity (*n* = 1), agitation (*n* = 3), new aggression (*n* = 1), one or more of the above (*n* = 5). ^2^ Seven dogs did not receive a “highest” dose; therefore, *n* was reduced. ^3^ *p*-value reported for exact McNemar chi-square test for paired observations comparing reported side effects of gabapentin at “lowest” dose as compared to “highest dose” (*n* = 43).

**Table 2:** Frequency of side effects of gabapentin based on the qualitatively reported “lowest dose” administered (range 3–25 mg/kg), further divided into two quantitative mg/kg ranges in 50 dogs with behavior disorders.

Side Effect ^1^	No. Dogs at Each Quantitative Dosing Category (%) with Owner Reported Side Effect		*p*-Value ^2^
	<15 mg/kg (*n* = 39)	15–30 mg/kg (*n* = 11)	
Sedation	4 (10.3%)	1 (10.0%)	1.0
Increased activity	3 (7.7%)	1 (10.0%)	1.0
Agitation	5 (12.8%)	1 (10.0%)	1.0
Increased appetite	1 (2.6%)	0	1.0
Urinary incontinence	0	1 (10.0%)	0.2
New aggression	2 (5.1%)	1 (10.0%)	0.5
One or more of the above	9 (23.1%)	4 (40.0%)	0.4

^1^ No side effects were reported for ataxia, vomiting, diarrhea, constipation, or increased aggression, which are therefore omitted from the table. ^2^ Fisher’s Exact Test.

**Table 3:** Frequency of side effects of gabapentin based on the qualitatively reported “highest dose” administered (range 13.0–65 mg/kg), further divided into four quantitative mg/kg ranges in 43 dogs with behavior disorders.

Side Effect ^1^	No. Dogs at Each Quantitative Dosing Category (%) with Owner Reported Side Effect				*p*-Value ^2^
	<15 mg/kg (*n* = 3)	15–30 mg/kg (*n* = 9)	30.1–49.9 mg/kg (*n* = 23)	≥50 mg/kg (*n* = 8)	
Sedation	1 (33.3%)	2 (22.2%)	13 (56.5%)	5 (62.5%)	0.3
Ataxia	0	3 (33.3%)	4 (17.4%)	2 (25.0%)	0.6
Increased activity	1 (33.3%)	1 (11.1%)	2 (8.7%)	0	0.4
Agitation	1 (33.3%)	3 (33.3%)	3 (13.0%)	1 (12.5%)	0.4
Increased appetite	0	1 (11.1%)	1 (4.3%)	1 (12.5%)	0.5
Diarrhea	0	0	1 (4.3%)	1 (12.5%)	0.5
Constipation	0	0	1 (4.3%)	0	1.0
Urinary incontinence	0	0	2 (8.7%)	0	1.0
Increased aggression	0	0	1 (4.3%)	0	1.0
One or more of the above	2 (66.7%)	5 (55.6%)	16 (69.6%)	6 (75.0%)	0.9

^1^ No side effects were reported for vomiting or new aggression, therefore are omitted from the table. ^2^ Fisher’s Exact Test.

**Table 4:** Diagnosis and owner reports that gabapentin was “effective” or “not effective” at improving their dog’s behavior.

Diagnosis	No. Owner Reported Effective for Dogs with Listed Diagnosis (%) ^1^	No. Owner Reported Effective for Dogs without Listed Diagnosis (%) ^1^	*p*-Value ^2^
Fear-related aggression	27 (75.0%)	9 (64.3%)	0.5
Generalized anxiety	24 (70.6%)	12 (75.0%)	1.0
Specific phobia	15 (68.2%)	21 (75.0%)	0.8
Conflict-related aggression	14 (93.3%)	22 (62.9%)	0.04
Noise phobia	8 (88.9%)	28 (68.3%)	0.4
Separation anxiety	5 (62.5%)	31 (73.8%)	0.7
Aggression secondary to arousal	2 (28.6%)	34 (79.1%)	0.01
Global fear	5 (83.3%)	31 (70.5%)	0.7

^1^ Owner reports categorized as “effective” (“very effective”, “moderately effective”) or “minimally/not effective” (“slightly effective”, “not effective”). ^2^ Fisher’s Exact Test comparing owner reported effective for listed diagnosis as compared to owners of dogs without that diagnosis.

## Data Availability

The data presented in this study are available on request from the corresponding author due to patient-client confidentiality.

## Supplementary Materials

The following supporting information can be downloaded at: https://www.mdpi.com/article/10.3390/ani14101462/s1. File S1: Retrospective Evaluation of Gabapentin in Dogs.

## Appendix A

The breeds represented amongst the purebred/purpose-bred dogs were the American Pitbull Terrier (*n* = 2), Basenji (*n* = 1), Bernedoodle (*n* = 1), Bernese Mountain Dog (*n* = 1), Bluetick Coonhound (*n* = 1), Boston Terrier (*n* = 1), Cavalier King Charles Spaniel (*n* = 1), French Bulldog (*n* = 2), Great Pyrenees (*n* = 1), Havanese (*n* = 1), Labradoodle (*n* = 1), Labrador Retriever (*n* = 1), Portuguese Water Dog (*n* = 2), Pug (*n* = 1), Shar Pei (*n* = 1), St. Bernard (*n* = 1), Tamaskan Dog (*n* = 1), and the Wheaton Terrier (*n* = 2).
