# Supplementary material for: Effects of Gabapentin on the Treatment of Behavioral Disorders in Dogs: A Retrospective Evaluation
Source: Animals (Basel). 2024 May 14;14(10):1462. doi: 10.3390/ani14101462 (PMC11117262; doi:10.3390/ani14101462)
Supplement: Supplementary file 1 [file animals-14-01462-s001.zip › animals-2982967-supplementary.pdf]

Massachusetts Veterinary Behavior

Retrospective Evaluation of Gabapentin in Dogs

1. **Contact Information**
   Owner Name (first and last):
   Dog Name:
   Email Address:
   Phone Number:

   Our records indicate that gabapentin was prescribed to your dog. Is this correct?
   Yes No

2. **Gabapentin Dose/Frequency**
   Did you try more than one dose of gabapentin?
   Yes No

   What is the highest and lowest dose of gabapentin (in mg) you have given (or if current, what is the dose you currently give)?
   Highest Dose (mg):
   Lowest Dose (mg):
   Current Dose (mg):

   Which dose of gabapentin (in mg) did you/do you most commonly use?

   Which of the following best describes how frequently you gave gabapentin to your dog?
   __Gabapentin was given every day
   __Gabapentin was only given as needed
   __I used this medication only 1-3 times
   __Other (please specify):

3. **Efficacy**
   What was the effectiveness of gabapentin when given to your dog? Would you describe it as:
   __Very effective
   __Moderately effective
   __Slightly effective
   __Not at all effective

4. **Side Effects**
   We would now like to ask about the side effects your dog experienced at the low dose and high doses of gabapentin.

For the following listed side effects, please answer yes or no as to whether your dog experienced that effect at the low and/or high dose (if you answered "yes" to the given side effect, please describe how much the side effect bothered you):

	Low Dose		High Dose		Did this side effect bother you?			
Side Effect	Yes	No	Yes	No	Not at all	Somewhat	Very much	N/A
Sedation (your dog was sleepy/tired from this medication)								
Unsteady or "drunk" (falling, stumbling, bumping into walls, etc.)								
Increased activity (running or jumping around, as though your dog had just had a very strong cup of coffee)								
Agitation (seemed irritable, restless, difficulty relaxing, vocalizing)								
Increased appetite								
Vomiting								
Diarrhea								
Constipation								
Urine Dribbling								
New aggression (meaning your dog growled, snapped, lunged, or bit people or other animals when under the influence of this drug when your dog had never previously shown aggression in any context)								
Increased aggression (meaning your dog growled, snapped, lunged, or bit a person or animal in a manner that is very uncharacteristic for this dog when not under the influence of this medication)								

Are you still administering gabapentin? If you answer "no" to the following question, please skip to section 5.

Yes   No

Why did you discontinue gabapentin (check all that apply)?

__Was no longer needed

__Was not effective

__Undesirable effects

__Other (please specify):

If you answered "undesirable effects" as the reason to discontinue gabapentin, please tell us the undesirable effects you witnessed (check all that apply):

__too sleepy

__too unsteady or "drunk"

__activity level too high
__caused or worsened aggression
__caused diarrhea
__caused constipation
__caused constant urine dribbling
__difficult to get dog to take pill
__appetite too high
__Other (please specify):
